# A stress-reduction eHealth intervention for healthcare workers: a pilot implementation study

**DOI:** 10.1192/j.eurpsy.2025.520

**Published:** 2025-08-26

**Authors:** B. García-Vázquez, C. Bayón Pérez, M. F. Bravo Ortiz, R. Mediavilla Torres

**Affiliations:** 1 Psychiatry, Clinical Psychology and Mental Health, Hospital Universitario La Paz (HULP); 2 Psychiatry, Instituto de Investigación Sanitaria La Paz (IdiPAZ); 3 Psychiatry, Universidad Autónoma de Madrid (UAM); 4 Centro de Investigación Biomédica en Red de Salud Mental (CIBERSAM); 5 Instituto de Investigación Carlos III; 6 Hospital Universitario La Princesa; 7 Instituto de Investigación Sanitaria del Hospital Universitario La Princesa, Madrid, Spain

## Abstract

**Introduction:**

Public health and policy require effective health strategies for diverse contexts. Despite numerous studies, a gap exists in translating knowledge to real-world scenarios. The RESPOND study’s stepped-care intervention has shown efficacy in anxiety and depression among healthcare workers, recognized by the European Commission as a best practice. Conducting a pilot trial in Madrid, we aimed to address this research-to-practice gap.

**Objectives:**

After evaluating a previous randomized controlled trial, we adapted our intervention for a primary care center in Madrid, implementing it over 5-6 weeks in November-December 2023. We used two strategies: remote delivery via app or website with weekly calls, and hybrid, with group sessions instead of calls.

**Methods:**

Gathering data included sociodemographic (age, gender, and type of job), clinical (Kessler Psychological Distress Scale, K-10), and qualitative insights through interviews, to explore implementation outcomes from Proctor’s model (Acceptability, Appropriateness, Feasibility, Fidelity, and some ideas for future large-scale implementation). We used mixed methods to analyse data. For the quantative data, we used descriptive statistics such as means, frequencies and percentages to describe the results. For qualitative data, interview transcriptions were analysed following thematic analysis guidelines.

**Results:**

We included 17 out of 39 healthcare workers (44%) working at the primary care center, the sample included 8 doctors, 5 medical residents, 3 nurses, and 1 social worker. Only 3 were male. Over half chose the hybrid option, with 2 dropouts. Regarding clinical information, 88% of participants initially reported psychological distress, but after the intervention, this dropped to 66%. Participants found the intervention timely and appropriate, with an average satisfaction rating of 8 out of 10.

**Conclusions:**

Preliminary findings show the intervention was well-received, and both strategies were successful, reaching a broad audience. Thematic analysis of the interviews is still ongoing. Preliminary results suggest that these strategies are appropriate /feasible for the Madrilenian healthcare system.

**Disclosure of Interest:**

None Declared

